# p-Type PVA/MWCNT-Sb_2_Te_3_ Composites for Application in Different Types of Flexible Thermoelectric Generators in Combination with n-Type PVA/MWCNT-Bi_2_Se_3_ Composites

**DOI:** 10.3390/polym14235130

**Published:** 2022-11-25

**Authors:** Jana Andzane, Krisjanis Buks, Juris Bitenieks, Lasma Bugovecka, Artis Kons, Remo Merijs-Meri, Janis Svirksts, Janis Zicans, Donats Erts

**Affiliations:** 1 Institute of Chemical Physics, University of Latvia, LV-1586 Riga, Latvia; 2 Institute of Polymer Materials, Faculty of Materials Science and Applied Chemistry, LV-1048 Riga, Latvia; 3 Faculty of Chemistry, University of Latvia, LV-1586 Riga, Latvia

**Keywords:** flexible thermoelectric composites, hybrid network, polyvinyl alcohol-based composite materials, multiwall carbon nanotubes, antimony telluride nanostructures, radial thermoelectric generator

## Abstract

This work is devoted to the fabrication of p-type polyvinyl alcohol (PVA)-based flexible thermoelectric composites using multiwall carbon nanotubes-antimony telluride (MWCNT-Sb_2_Te_3_) hybrid filler, the study of the thermoelectrical and mechanical properties of these composites, and the application of these composites in two types (planar and radial) of thermoelectric generators (TEG) in combination with the previously reported PVA/MWCNT-Bi_2_Se_3_ flexible thermoelectric composites. While the power factors of PVA/MWCNT-Sb_2_Te_3_ and PVA/MWCNT-Bi_2_Se_3_ composites with 15 wt.% filler were found to be similar, the PVA/MWCNT-Sb_2_Te_3_ composite with 25 wt.% filler showed a ~2 times higher power factor in comparison with the PVA/MWCNT-Bi_2_Se_3_ composites with 30 wt.% filler, which is attributed to its reduced electrical resistivity. In addition, developed PVA/MWCNT-Sb_2_Te_3_ composites showed a superior mechanical, electrical, and thermoelectric stability during 100 consequent bending cycles down to a 3 mm radius, with insignificant fluctuations of the resistance within 0.01% of the initial resistance value of the not bent sample. Demonstrated for the first time, 2-leg TEGs composed from p-type PVA/MWCNT-Sb_2_Te_3_ and n-type PVA/MWCNT-Bi_2_Se_3_ composites showed a stable performance under different external loads and showed their potential for applications involving low temperature gradients and power requirements in the range of nW.

## 1. Introduction

The development of flexible thermoelectric devices is an emerging trend in the field of thermoelectrics because of their high potential in conversion to usable energy waste heat released by differently shaped surfaces, as well as in applications in self powering sensors and wearable devices [1]. The performance of thermoelectric devices is characterized by a dimensionless figure of merit ZT, defined as S^2^·*ρ*^−1^·κ^−1^·T, where S is the Seebeck coefficient of the material, *ρ* is its electrical resistivity, T is absolute temperature, and κ is the thermal conductivity of the material. S^2^·*ρ*^−1^ is usually referred to as the power factor (PF) of thermoelectric material. Generally, the thermoelectric performance of the material can be improved by the reduction in its thermal conductivity without affecting its electrical resistivity. The common approach for the reduction in the thermal conductivity of the material is reduction in its dimensions down to the nanoscale, when effective phonon scattering occurs at the surfaces of the nanostructures, as well as at the grain boundaries and interfaces between the nanostructures [2,3]. 

For the development of an efficient thermoelectric generator (TEG), thermoelectric materials with both p-type and n-type conductivities are required. The main architecture used to develop TEG from rigid inorganic materials is planar configuration, where p-type and n-type legs are arranged on the planar substrate and interconnected with electrodes [4]. However, for applications involving a low-grade heat conversion (for example, wearable electronics powered by the heat produced by a human body and the conversion of domestic heat from hot pipes, etc.), flexible TEGs that can be adjusted to the shape of the heated surface should be developed. In the case of using planar configuration for flexible thermoelectric devices, the rectangular strips of p- and n-type thin inorganic films [2] or composite film legs [4] of the TEG are deposited on flexible substrates with pre-fabricated electrodes. Recently, a promising innovative radial configuration of TEG, where p- and n-type disk-shaped legs are surrounding the cylindrical heat source (for example, the hot pipe) was proposed for the flexible thermoelectric composite materials [1,5]. 

Nowadays, the most promising materials for the application in flexible thermoelectrics are composites prepared from a mixture of electrically conductive polymers in combination with inorganic nanostructured thermoelectric filler as, for example, carbon nanotubes, tellurium nanowires, bismuth, and antimony chalcogenides nanoparticles, etc. [1]. The reported power output for flexible conductive polymer-inorganic filler TEGs is in the range from ~17 nW·cm^−2^ at ΔT = 20 K up to 50 μW·cm^−2^ at ΔT = 70 K [1]. However, despite the satisfying efficiency of the composites based on electrically conductive polymers with different fillers, the usage of electrically conductive polymer matrixes has some significant drawbacks. Since most conductive polymers require oxidative doping, the properties of the resulting state are crucial. Such materials are salt-like (polymer salt), which diminishes their solubility in organic solvents and water and, hence, their processability. Furthermore, the charged organic backbone is often unstable towards atmospheric moisture. The poor processability for many polymers requires the introduction of solubilizing or substituents, which can further complicate the synthesis. Experimental and theoretical thermodynamical evidence suggests that conductive polymers may even be completely and principally insoluble so that they can only be processed by dispersion [6].

An alternative and more environmentally friendly solution for the fabrication of flexible thermoelectric composites is the use of a non-conductive polymer matrix as, for example, poly(vinyl)alcohol (PVA). PVA is a suitable matrix material due to its solubility in water which alleviates the preparation of thermoelectric composites without using hazardous organic solvents. While properties of PVA-based composites are widely studied and used for the applications in optical sensors, packaging, filter materials, battery applications, biomedical scaffolds, etc. [7], the study of their applicability in flexible thermoelectrics is limited to a few reports [8,9]. Previous research showed that despite the solubility of PVA in water, the PVA-based n-type thermoelectric composites show stable reversible behavior under relative humidity (RH) levels up to 60% [10]. As it is known, RH levels between 30 and 60% are optimal for health, work performance, and a lower risk of infection for humans [11], and are generally recommended for air-conditioned buildings. Thus, the PVA-based thermoelectric composites are suitable candidates for indoors applications related to low-grade heat conversion (for example, the conversion of human body heat or hot pipes). In addition, some previous reports showed that the application of PVA as a binder for thermoelectric nanomaterials may noticeably enhance their Seebeck coefficient [12,13]. The main reported drawback of the use of PVA as a matrix for thermoelectric composites is the very high electrical resistivity of the neat PVA, and consequently, the output power of the resulting thermoelectric composites, which are significantly lower in comparison with the electrically conductive polymers-based counterparts. For example, a PVA/Bi_2_Te_3_ composite with a filler content of 90% of the total composite mass reached the maximum output power of 9 μW·cm^−2^ at ΔT = 46 K [8]. It should be noted that bismuth and antimony chalcogenides (Bi_2_Se_3_, Bi_2_Te_3_, and Sb_2_Te_3_) are the most perspective inorganic fillers for the polymer-based composites. These materials are known as the best candidates for near-room temperature thermoelectric applications [14]. In contrast with those widely used as thermoelectric fillers carbon nanotubes, which are naturally p-type and require an additional processing to obtain and maintain the n-type conductance, bismuth and antimony chalcogenides are naturally n-type (Bi_2_Se_3_, Bi_2_Te_3_) and p-type (Sb_2_Te_3_) materials. However, due to the high proportions (up to 90 wt.% of total composite mass) of these thermoelectric fillers which are required for obtaining satisfying thermoelectric properties, the resulting composite loses a lot of its flexibility. To improve the performance of the bismuth and antimony chalcogenides as the thermoelectric fillers, these materials can be grown in a form of crystalline nanostructures or nanostructured thin films directly on the surfaces of graphene or carbon nanotubes [15,16]. Due to the direct electrical contact between the graphene or carbon nanotubes and inorganic nanostructures, established during the synthesis, such hybrid nanostructured materials become flexible, while retaining a high electrical conductance and Seebeck coefficients comparable with their bulk inorganic counterparts [16]. Recently, our group reported the application of such n-type multiwall carbon nanotube-bismuth selenide (MWCNT-Bi_2_Se_3_) hybrid networks as a filler for n-type PVA-based thermoelectric composites [10,17]. These composites reached PF values significantly exceeding the PFs reported by Pires et al. for n-type PVA/Bi_2_Te_3_ composites [8], while requiring three times less (15–30 wt.% vs. 60–90 wt.%) filler material. In addition, these composites demonstrated a good mechanical and electrical stability over the 100 consequent bending cycles down to a 10 mm radius. 

In this work, the fabrication of p-type flexible PVA-based thermoelectric composites, employing MWCNT-Sb_2_Te_3_ hybrid networks as a thermoelectric filler, is presented and their thermoelectric properties are studied. Planar and radial 2-legs TEGs, consisting of previously reported n-type PVA/MWCNT-Bi_2_Se_3_ [17] and p-type PVA/MWCNT-Sb_2_Te_3_ composites, are demonstrated and characterized for the first time to the best of our knowledge. 

## 2. Materials and Methods

As a polymer matrix was used, PVA (Celvol E 04/88S) with the degree of hydrolysis of 88% (Celanese Corporation, Irving, TX, USA) and as a basis for thermoelectric filler preparation, we used commercial MWCNTs with an outer mean diameter of 13 nm, inner mean diameter of 4 nm, bulk density of 130–150 kg·m^−3^, and a purity of 95% (Baytubes^®^ C150 P, Bayer MaterialScience, Covestro, Leverkusen, Germany), as well as laboratory-synthesized hybrid networks of MWCNT-Sb_2_Te_3_ (MWCNT:Sb_2_Te_3_ mass ratio 65:35). The MWCNT-Sb_2_Te_3_ hybrid networks were prepared by catalyst-free physical vapor deposition of Sb_2_Te_3_ (99.999%, CAS: 1327-50-0, Alfa Aesar, Kandel, Germany) on MWCNT substrates. MWCNT substrates were prepared by the spray-coating technique as described elsewhere [16]. The wt% of the MWCNTs in the MWCNT-Sb_2_Te_3_ hybrid networks was determined by weighing the substrate components using analytical scales (XR 205SM-DR, Precisa, Dietikon, Switzerland) at each stage of the preparation of the hybrid networks. The thermoelectric PVA composites were prepared by dissolving pristine PVA in de-ionized water at 80 °C by mixing with magnetic stirrer on a hot plate for ~30 min. Then, MWCNT-Sb_2_Te_3_ thermoelectric filler was dispersed in a prepared PVA solution by an ultrasonic sonicator UIS250V (Hielscher Ultrasonics GmbH, Teltow, Germany) for 20 min to achieve the homogenous dispersion of the thermoelectric filler. Free-standing films of PVA/MWCNT-Sb_2_Te_3_ composites for the characterization and TEG fabrication were obtained by solution casting on a flat polypropylene surface. The drying time of the PVA/MWCNT-Sb_2_Te_3_ composite films was for 24 h at an ambient temperature.

The morphological characterization of the samples was performed by a field-emission scanning electron microscope (S-4800, Hitachi, Tokyo, Japan), equipped with an energy-dispersive X-ray diffraction analyzer (Bruker XFlash Quad 5040, Billerica, MA, USA), transmission electron microscope (FET Technai GF 20, FEI Company, Hillsboro, OR, USA), and X-ray diffractometer (XRD, Bruker D8 Discover, Billerica, MA, USA). For the identification of the diffraction peaks, the ICDD database PDF-2/Release 2021 (Ref. cards PDF 01-085-4141 (Sb_2_Te_3_), PDF 00-001-0727 (Te), and PDF 01-075-1565 Sb_2_O_3_) was used.

The dynamic mechanical thermal properties and thermomechanical characteristics of the PVA-based composites were determined in tensile mode using a dynamic mechanical thermal analyzer Mettler Toledo DMA/SDTA861 and TMA/SDTA841 device (Mettler Toledo Inc., Cleveland, OH, USA), as described elsewhere [17].

The thermoelectrical measurements were carried out at room temperature (300 K) under ambient conditions using a home-made device, allowing for the bending of the samples which were calibrated by Standard Reference Material 3451 (NIST, Gaithersburg, MD, USA) for a low-temperature Seebeck coefficient, as described elsewhere [10]. The room-temperature electrical characterization was performed using the Keithley 6430 Sub-Femtoamp Remote Source Meter (Cleveland, OH, USA). The low-temperature resistance measurements were performed using the thermal transport option of the physical property measurement system DynaCool9T (Quantum Design, San Diego, CA, USA) and MultiVu software, as described elsewhere [10]. During the bending experiments, the resistance at a specific bending radius was calculated from a voltage setting (always 0.1 V ± 1 mV) and measured current (±100 pA). For each value of the bending radius, the number of measurements was between 9 and 16. The resistance of the sample during each measurement was calculated as the ratio of the voltage to the current. Then, the average resistance was calculated. For the durability experiments, similar measurements were made after each bending cycle (bending and straightening the sample). Then, the average resistance after a specific number (from 12 to 31) of bending cycles was calculated. The normalization of data was performed by dividing every average resistance value by the average resistance value of the not-bent film. The Seebeck coefficient was calculated from the measured voltage (±1 nA) and temperature (±0.01 K) for all the data. Then, the average Seebeck coefficient after a specific number (from 32 to 45) of bending cycles was calculated. The normalization of the data was performed by dividing every average Seebeck coefficient by the average Seebeck coefficient of the not-bent film. The standard deviation (SD) for the average resistance and average Seebeck coefficient was calculated using the Excel for Microsoft 365 function STDEV.S.

For the fabrication of the TEGs, PVA/MWCNT-Bi_2_Se_3_ composites with 30 wt.% filler and PVA/MWCNT-Sb_2_Te_3_ composites with 25 wt.% filler were used. Electrically conductive adhesive (EPO-TEK^®^H20E, Epoxy technology, Inc., Billerica, MA, USA) was used to apply the electrodes. The assembling of the planar and radial TEGs is described in detail in the Results and Discussion section. The voltage, which was thermally generated by the TEGs, was measured using a laboratory-built device under applied temperature gradients of 8–20 K for the planar TEG and 27–77 K for the radial TEG. For the characterization of the load-dependent behavior of the TEGs, external resistances of 30 kΩ–2 MΩ for the planar TEG and 5 kΩ–560 kΩ for the radial TEG were used. 

## 3. Results and Discussion

**Figure 1:**
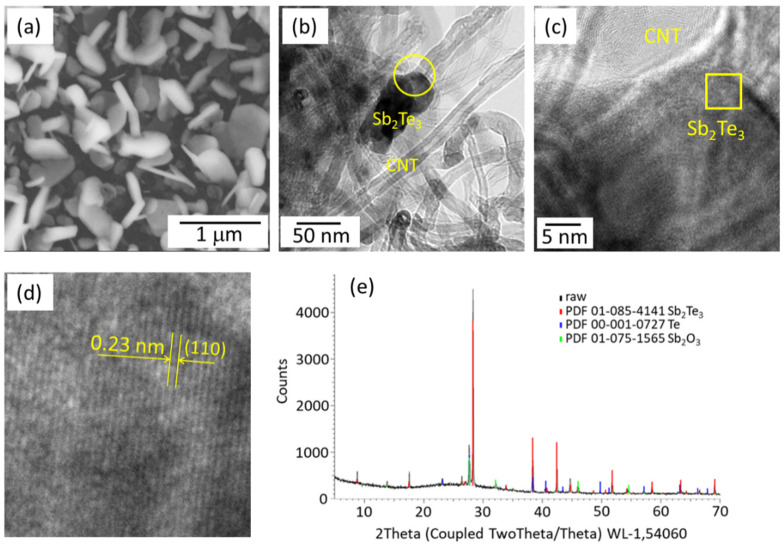
(**a**) scanning electron microscope image of MWCNT-Sb_2_Te_3_ hybrid network; (**b**–**d**) high-resolution transmission electron microscope images of MWCNT-Sb_2_Te_3_ hybrid network: low-magnification image (**b**), close look at the area labelled by the circuit (**c**), high-magnification image of the crystalline structure of Sb_2_Te_3_ cluster in the area labeled by square (**d**), (**e**) X-ray diffraction pattern of deposited Sb_2_Te_3_ nanostructures.

The Sb_2_Te_3_ (65 wt.%)-MWCNT (35 wt.%) hybrid structures (Figure 1a) synthesized as described elsewhere [16] were used as the filler for the preparation of the PVA-MWCNT-Sb_2_Te_3_ composites with filler mass fractions of 10, 15, and 25 wt.%. Such filler concentrations were chosen to obtain electrically conductive samples, but at the same time to avoid the unwanted agglomeration of the filler, observed previously for the PVA-based composites at hybrid filler concentration of 30 wt.% [17]. As previously reported by our group, the Sb_2_Te_3_ deposits on the MWCNT network in cluster form [16]. A representative image of a Sb_2_Te_3_ cluster formed on the MWCNT network reveals the single-crystalline nature of Sb_2_Te_3_ with an interplanar distance 0.23 Å corresponding to [110] crystallographic growth direction (Figure 1b–d). The chemical composition of deposited Sb_2_Te_3_ nanostructures is proved by the EDX measurements performed for the as-grown MWCNT-Sb_2_Te_3_ hybrid networks, showing a Sb:Te ratio of (38 ± 3):(62 ± 4) at.%, which corresponds to the stoichiometric Sb_2_Te_3_ compound. The XRD pattern of deposited Sb_2_Te_3_ showed the presence and prevalence of all peaks, which are characteristic of powder Sb_2_Te_3_, indicating the random orientation of the Sb_2_Te_3_ nanostructures (Figure 1e, red curve). The presence of ~12% of antimony oxide Sb_2_O_3_ (Figure 1e, green curve) is consistent with the previously reported data for Sb_2_Te_3_ ultrathin films deposited on graphene [2], and indicates that the surfaces of the Sb_2_Te_3_ nanostructures are covered with the native oxide layer. In addition, the presence of an insignificant amount of tellurium was detected (Figure 1e, blue curve). This means the possible inclusion of Te clusters in MWCNT-Sb_2_Te_3_ hybrid structures. 

**Figure 2:**
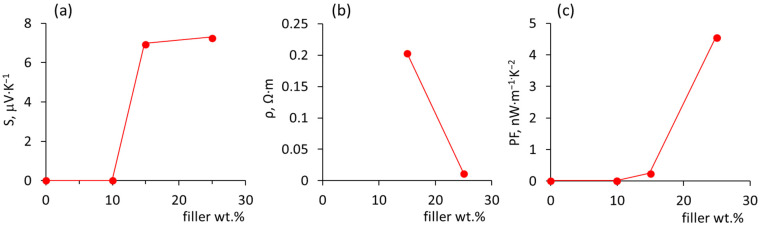
Comparison of the properties of the PVA/MWCNT-Sb_2_Te_3_ with10 wt.%, 15 wt.%, and 25 wt.% filler: (**a**) Seebeck coefficient; (**b**) electrical resistivity; (**c**) power factor.

The mass ratio 65 (Sb_2_Te_3_):35 (MWCNT) wt.% was chosen as it has been shown previously that as-deposited on a glass substrate MWCNT-Sb_2_Te_3_ hybrid structures with such a ratio showed a Seebeck coefficient of ~60 μV/K at room temperature (RT) [16], which is comparable with the RT Seebeck coefficient reported for the bulk Sb_2_Te_3_ (~70 μV·K^−1^) [18] and nanostructured Sb_2_Te_3_ thin films grown by catalyst-free transport (~75 μV·K^−1^) [19]. Simultaneously, the 35 wt.% mass fraction of MWCNTs in the MWCNT-Sb_2_Te_3_ hybrid structures was used as a filler for PVA/MWCNT-Sb_2_Te_3_ composited with 10, 15 and 25 wt.% MWCNT-Sb_2_Te_3_ filler means that the mass fractions of the MWCNTs in the resulting composites will be ~3.6%, 5.5%, and ~9%, respectively, which should be enough for the formation of the electrically conductive network in the PVA matrix, connecting together Sb_2_Te_3_ nanostructures, without an undesired agglomeration [20,21]. However, it was found out that the PVA/MWCNT-Sb_2_Te_3_ composite with 10 wt.% filler remains non-conductive. In turn, PVA/MWCNT-Sb_2_Te_3_ composites with 15 and 25 wt.% filler (i.e., with 5.5 and 9 wt.% of MWCNTs, respectively) showed positive Seebeck coefficients of ~6.9 and 7.2 μV·K^−1^ (Table 1, Figure 2a).

However, at the same time, the absolute value of the Seebeck coefficient of PVA/MWCNT-Sb_2_Te_3_ is by an order of magnitude lower than the absolute value of the Seebeck coefficient shown by the previously reported PVA/MWCNT-Bi_2_Se_3_ composites [17], as well as of the Seebeck coefficient shown by as-grown MWCNT-Sb_2_Te_3_ hybrid networks [16] (Table 1). Presumably, the lower values of the Seebeck coefficient shown by the PVA/MWCNT-Sb_2_Te_3_ composites are related to the difference in the growth mechanisms of Sb_2_Te_3_ and Bi_2_Se_3_ on the surface of the MWCNT network [16]. In contrast with Bi_2_Se_3_, which forms core–shell structures with the MWCNTs with the following formation of free-standing Bi_2_Se_3_ nanoplates, starting from the core–shell structures, Sb_2_Te_3_ deposits on the MWCNT network in nanoclusters (Figure 1a,b). When MWCNT-Bi_2_Se_3_/Sb_2_Te_3_ hybrid networks are scratched off the glass substrate and mechanically mixed with the PVA, the established during the hybrid network growth connections between the thermoelectric nanostructures, as well as interfaces between the thermoelectric material and MWCNTs, may be weakened and partially broken during the mixing process, and a new electrically conductive network from the MWCNT-Bi_2_Se_3_/Sb_2_Te_3_ hybrid structures throughout the PVA matrix is formed. However, the nanocluster form of Sb_2_Te_3_ leads to the separation of these nanostructures during the preparation of the composite. As a result, a nanostructured Sb_2_Te_3_ network with a smaller number of connections is formed in the PVA matrix compared to the as-grown MWCNT-Sb_2_Te_3_ network.

PVA/MWCNT-Sb_2_Te_3_ composites with 15 and 25 wt.% filler were found to be electrically conductive, showing an electrical resistivity of 0.2 Ω·m and 0.01 Ω·m, respectively, which is significantly higher than the electrical resistivity of the previously reported n-type PVA/MWCNT-Bi_2_Se_3_ composites with a filler content of 15 and 30 wt.% (11.5 Ω·m and 4.8 Ω·m, Table 1, Figure 2b). Presumably, a lower electrical resistivity of PVA/MWCNT-Sb_2_Te_3_ composites is due to the higher concentration of MWCNTs in the composites in comparison with the PVA/MWCNT-Bi_2_Se_3_ composites (Table 1). Despite the relatively low Seebeck coefficient, the PF of the PVA/MWCNT-Sb_2_Te_3_ composite with 15 wt.% filler was comparable with the PF of the PVA/MWCNT-Bi_2_Se_3_ composite with the same concentration of filler (0.24 nW·m^−1^·K^−2^ and 0.28 nW·m^−1^·K^−2^, respectively, Table 1, Figure 2c). In turn, the PF of the PVA/MWCNT-Sb_2_Te_3_ composite with 25 wt.% was ~2 times higher than the PF of PVA/MWCNT-Bi_2_Se_3_ with 30% of filler (4.6 nW·m^−1^·K^−2^ vs. 2.5 nW·m^−1^·K^−2^, Table 1, Figure 2c). 

**Figure 3:**
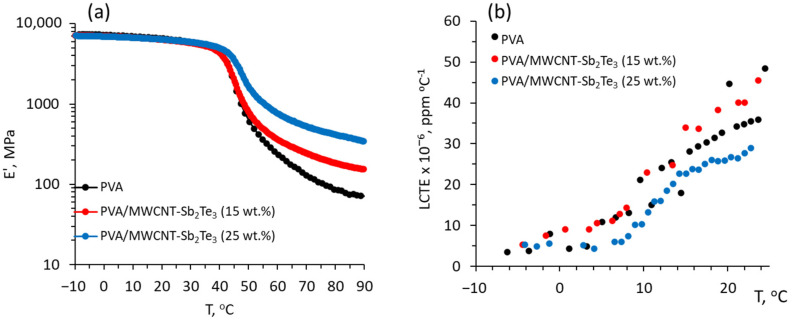
(**a**) storage modulus and (**b**) linear coefficient of thermal expansion (LCTE) vs. temperature of neat PVA and PVA/MWCNT-Sb_2_Te_3_ composites.

In addition, the study of the storage modulus of the PVA/MWCNT-Sb_2_Te_3_ composites in the region above the glass transition temperature showed as being noticeably higher in comparison with the neat PVA, and increasing with the increase in the filler content in the composite (Figure 3a). 

These results indicate a significant strengthening of the composite by the addition of the MWCNT-Sb_2_Te_3_ filler and indirectly prove the absence of an undesirable partial agglomeration of the filler at concentrations up to 25 wt.%. Previously, the partial agglomeration of the filler, resulting in a reduction in the storage modulus down to the values of neat PVA, had been observed in PVA/MWCNT-Bi_2_Se_3_ composites with a filler content of 30 wt.% [17]. PVA/MWCNT-Sb_2_Te_3_ composite with 25 wt.% filler showed also a noticeably lower linear coefficient of thermal expansion (LCTE) in comparison with the neat PVA and PVA/MWCNT-Sb_2_Te_3_ composite with 15 wt.% filler (Figure 3b). Presumably, such an improvement of the dimensional stability of the composite is related to the formation of a filler network throughout the PVA matrix, which restricts the macromolecular movement [22]. 

**Figure 4:**
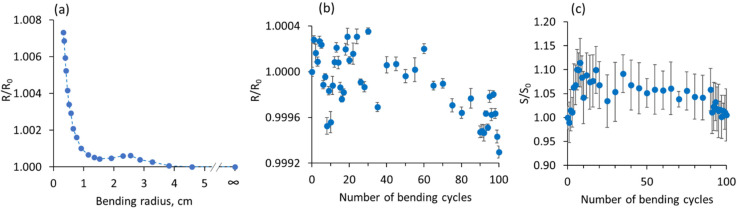
Bending tests of PVA/MWCNT-Sb_2_Te_3_ composite with 25 wt.% filler: (**a**) relative changes in the resistance vs. its bending radius, and (**b**) relative changes in the resistance during 100 consequent bending cycles down to 0.3 cm (R is the resistance of bent composite, R_0_ is the resistance of the composite before the first bending cycle); (**c**) relative changes of the Seebeck coefficient of the composite during 100 consequent bending cycles down to 0.3 cm (S is the Seebeck coefficient of bent composite, S_0_ is the Seebeck coefficient of the composite before the first bending cycle). The error bars represent the standard deviation (SD) for average resistance (**b**) and average Seebeck coefficient (**c**).

The bending tests performed at RT for the PVA/MWCNT-Sb_2_Te_3_ composite with 25 wt.% filler showed that the changes in the resistance of this composite upon bending down to the radius of ~1 cm are negligible (less than 0.1%, Figure 4a). 

The subsequent bending of the composite to a radius of 0.3 cm resulted in a rapid increase in the resistance of the sample. However, the maximum relative change in the resistance still was no more than 1%. This is ~6.5 times smaller in comparison with the previously observed bending behavior of PVA/MWCNT-Bi_2_Se_3_ composites [17]. In addition, in contrast with the previously reported bending behavior of the PVA/MWCNT-Bi_2_Se_3_ composite, where an increase in the resistance by ~25% was observed during the first 20 bending cycles [17] down to a 3 mm radius, the resistance of the PVA/MWCNT-Sb_2_Te_3_ remained nearly constant during 100 consequent bending cycles (Figure 4b), with negligible fluctuations within 0.01% of the initial resistance value of the not bent sample. Presumably, the high electrical stability of the PVA/MWCNT-Sb_2_Te_3_ composites is related to the higher content of MWCNTs in them in comparison with the MWCNT content in PVA/MWCNT-Bi_2_Se_3_ composites (5.5–9 wt.% vs. 1.5–3 wt.%, Table 1), because at higher MWCNT concentrations, the partial disruption of the interconnections between them during the bending does not have a significant impact on the electrical conductance of the sample. The Seebeck coefficient of the PVA/MWCNT-Sb_2_Te_3_ composite with 25 wt.% filler showed an increase of ~5–10% during the first 20 bending cycles and remained at a constant level with insignificant fluctuations for the next 70 bending cycles, followed by the return to the value of the initial Seebeck coefficient of the not-bent sample during the last 10 bending cycles (Figure 4c). The increase in the Seebeck coefficient may be related to the establishment of new interconnects in the MWCNT-Sb_2_Te_3_ network inside the PVA matrix, which were durable during ~70 consequent bending cycles.

**Figure 5:**
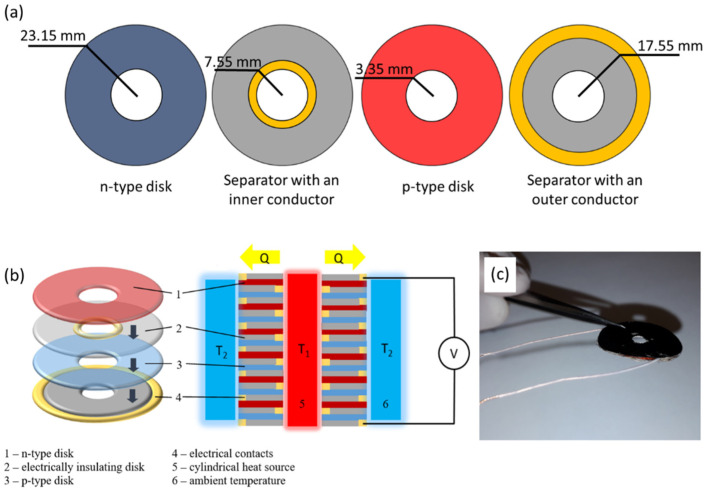
Radial thermoelectric generator (TEG): (**a**) schematics and dimensions of the radial disks; (**b**) schematics of the 2-leg radial TEG; (**c**) an image of the assembled PVA composites-based 2-leg TEG.

Composites with the best PF (p-type PVA/MWCNT-Sb_2_Te_3_ with 25 wt.% filler and n-type PVA/MWCNT-Bi_2_Se_3_ with 30 wt.% filler) were used for the fabrication of two types of thermoelectric generators: 2-leg planar TEG and 2-leg radial TEG. For the fabrication of the 2-leg planar TEG, the 10 mm × 15 mm large strips of PVA/MWCNT-Bi_2_Se_3_ composites with 30 wt.% filler and PVA/MWCNT-Sb_2_Te_3_ composites with 25 wt.% filler were used. Electrically conductive adhesive was used to apply the electrodes along the 15 mm long sides of the composite strips and to attach the copper connection wires. For the fabrication of a 2-leg radial TEG, disk shaped p-type, n-type, copper, and PVA elements were stamped out by using lathed stainless-steel rods. The inner diameter of the radial symmetry prototype device is 3.35 mm, the outer diameter being 23.15 mm. The outer diameter of the inner conductive copper disks is 7.55 mm, and the inner diameter of the outer conductive copper disks is 17.55 mm (Figure 5a).

The elements are coaxially stacked in layers to form a cylindrical geometry. PVA layers, located between the p- and n-type elements, are electrically insulating them and provide electrically conductive copper traces (radially between the p- and n-type disks and axially between the p- and n-type disks and the insulating layer) connected in the series. The conductive trace connections are done by using a conductive silver paint, however, the insulating connections are done by using an insulating adhesive (Figure 5b,c).

**Figure 6:**
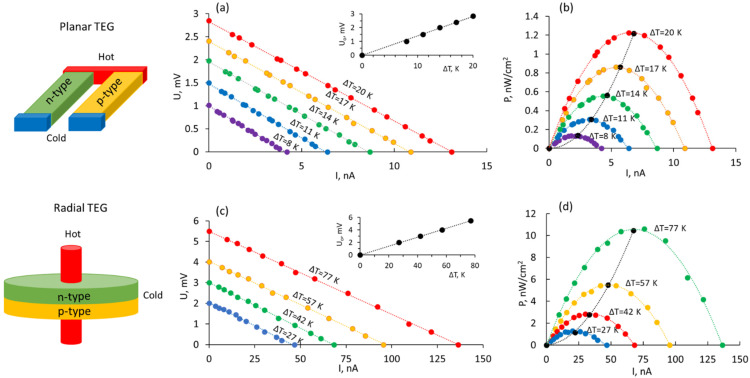
Characterization of load-dependent behavior of the PVA composites-based TEG prototypes: (**a**,**c**) output voltage vs. output current (insets—thermally generated open circuit voltage vs. applied temperature gradient); (**b**,**d**) output power per cross-section unit area vs. output current (black dots represent maximal power for each temperature gradient).

The performance of TEGs was characterized by determining the external load-dependence behavior. The open-circuit voltages generated by TEGs (insets in Figure 6a,c) showed linear dependencies on the temperature gradient that can be described by functions U_o_ = 0.15ΔT, reaching U_o_ = 3 mV at ΔT = 20 K for the planar TEG, and U_o_ = 0.07ΔT for radial TEG, reaching U_o_ = 1.4 mV at ΔT = 20 K.

These data mean that the performance of the radial TEG is ~2 times poorer in comparison with the planar TEG, presumably due to the higher heat dissipation from the surfaces of radial legs in comparison with the planar configuration, as well as the absence of controlled cold sink, which results in the more rapid equalization of the temperature gradient between the hot core and the outer edges of the radial device. At the same time, both types of TEGs showed linear and of equal slope dependencies of the output voltage vs. output current at different external resistances connected in series (Figure 6a,c), proving the stable operation of the devices and allowing to calculate the output power vs. the output current (Figure 6b,d). It was found that the maximal output power produced by both types of TEGs shows a power-law increase with the increase in the temperature gradient applied to the device (Figure 6b,d, black dots), and achieved a maximal power output of 1.2 nW·cm^−2^ at ΔT = 20 K for the two-leg planar TEG and 10 nW·cm^−2^ for the ΔT = 77 K for the two-leg radial TEG. Considering these results, the application of planar PVA composites-based devices is envisaged for the applications involving low temperature gradients and power requirements in the range of nW (for example, health environmental monitoring). In turn, radial TEG devices with the increased number of leg pairs can be used for the domestic waste heat conversion applications, for example, from the hot pipes.

## 4. Conclusions

PVA/MWCNT-Sb_2_Te_3_ flexible thermoelectric composites with a filler content of 25 wt.%, fabricated by the mechanical mixing of PVA with MWCNT-Sb_2_Te_3_ filler showed a p-type thermoelectric performance with the PF 4.6 nW·m^−1^·K^−2^, exceeding the PF of previously reported n-type PVA/MWCNT-Bi_2_Se_3_ composite with a 30 wt.% filler content by ~2 times. The bending tests during the 100 consequent bending cycles down to a 3 mm radius of the PVA/MWCNT-Sb_2_Te_3_ composite showed its superior mechanical, electrical, and thermoelectric stability, with the resistance fluctuations being within 0.01% and the Seebeck coefficient increasing by 5–10% during the first 20 cycles with a further stabilization at this level for the next 70 cycles. In addition, the PVA/MWCNT-Sb_2_Te_3_ composite with 25% filler showed a significant increase in the storage modulus and decrease in the linear coefficient of the thermal expansion in comparison with the neat PVA, which is attributed to the impact of the MWCNT-Sb_2_Te_3_ network established inside the PVA matrix. The 2-leg planar and radial TEGs, composed from the p-type PVA/MWCNT-Sb_2_Te_3_ composite with 25 wt.% filler and n-type PVA/MWCNT-Bi_2_Se_3_ composite with 30 wt.% filler showed a stable thermoelectric performance under different external electrical loads. The maximal thermally generated voltage shown by the demonstrated TEGs was 3 mV at ΔT = 20 K for the planar TEG, and 5.5 mV at ΔT = 77 K for the radial TEG. The poorer performance of the radial TEG was attributed to the excessive heat dissipation from the surfaces of the disk-shaped legs. Both types of PVA composites-based TEGs open the path for the development of thermoelectric devices operating at low temperature gradients (for example, the conversion of the heat from domestic hot pipes) and powering the processes, operating at the nW power range (for example, via environmental health monitoring).

## Figures and Tables

**Table 1:** Comparison of electrical resistivity, Seebeck coefficient and power factor of PVA-based composites with different types of fillers.

Sample	Filler wt.%	MWCNT wt.%	S, μV·K^−1^	*ρ*, Ω·m	PF, nW·m^−1^·K^−2^
PVA/MWCNT-Sb_2_Te_3_	15	5.5	6.9 ± 0.02	0.21 ± 0.01	0.24 ± 0.03
	25	9	7.2 ± 0.03	0.011 ± 0.001	4.6 ± 0.2
PVA/MWCNT-Bi_2_Se_3_ [7]	15	1.5	−60 ± 5	11.5 ± 0.5	0.28 ± 0.04
	30	3	−100 ± 4	4.8 ± 1.5	2.5 ± 1.1

## Data Availability

The data presented in this study are available on request from the corresponding author. The data are not publicly available as they are part of ongoing research.
